# Effects of tiotropium + olodaterol versus tiotropium or placebo by COPD disease severity and previous treatment history in the OTEMTO® studies

**DOI:** 10.1186/s12931-016-0387-7

**Published:** 2016-06-18

**Authors:** Dave Singh, Mina Gaga, Olaf Schmidt, Leif Bjermer, Lars Grönke, Florian Voß, Gary T. Ferguson

**Affiliations:** Centre for Respiratory Medicine and Allergy, The Medicines Evaluation Unit, University Hospital of South Manchester Foundation Trust, University of Manchester, Manchester, Southmoor Road, Manchester M23 9QZ, UK; Athens Chest Hospital, Athens, Greece; Lungen- und Bronchialheilkunde, Koblenz, Germany; Department of Respiratory Medicine and Allergology, Lund University, Lund, Sweden; Boehringer Ingelheim Pharma GmbH & Co. KG, Ingelheim, Germany; Pulmonary Research Institute of Southeast Michigan, Farmington Hills, MI USA

**Keywords:** COPD, Long-acting bronchodilator, Tiotropium, Olodaterol, Severity, Treatment history

## Abstract

**Background:**

As lung function declines rapidly in the early stages of chronic obstructive pulmonary disease (COPD), the effects of bronchodilators in patients with moderate disease and those who have not previously received maintenance therapy are of interest. OTEMTO® 1 and 2 were two replicate, 12-week, Phase III studies investigating the benefit of tiotropium + olodaterol on lung function and quality of life in patients with moderate to severe disease. *Post hoc* analyses were performed to assess the benefits for patients according to disease severity and treatment history.

**Methods:**

Four subgroup analyses were performed: Global initiative for chronic Obstructive Lung Disease (GOLD) 2/3, GOLD A/B/C/D, treatment naive/not treatment naive and receiving inhaled corticosteroids (ICS) at baseline/not receiving ICS at baseline. Primary end points were change in forced expiratory volume in 1 s (FEV_1_) area under the curve from 0 to 3 h response, change in trough FEV_1_ and St George’s Respiratory Questionnaire (SGRQ) total score. Transition Dyspnoea Index (TDI) focal score was a secondary end point, and SGRQ and TDI responder analyses were further end points; all were assessed at 12 weeks.

**Results:**

In all subgroups, patients receiving tiotropium + olodaterol responded better overall than those receiving tiotropium monotherapy. Improvements with tiotropium + olodaterol over placebo or tiotropium monotherapy were noted across GOLD 2/3 and GOLD A/B/C/D; however, improvements in SGRQ total score were most evident in the GOLD B subgroup. Moreover, lung-function outcomes were generally greater in those patients who had been receiving previous long-acting bronchodilator and/or ICS maintenance treatment.

**Conclusions:**

These data suggest that tiotropium + olodaterol should be considered as a treatment option in patients with moderate COPD who are initiating maintenance therapy, as well as those with more severe disease.

**Trial registration:**

ClinicalTrials.gov: NCT01964352 and NCT02006732.

**Electronic supplementary material:**

The online version of this article (doi:10.1186/s12931-016-0387-7) contains supplementary material, which is available to authorized users.

## Background

The use of long-acting β_2_-agonists (LABAs) and long-acting muscarinic antagonists (LAMAs) is central to the pharmacological management of patients with chronic obstructive pulmonary disease (COPD) [1, 2]. The aim of treatment is to improve lung function, reduce symptoms and risk of exacerbations, and improve health status [1].

Tiotropium is an established once-daily LAMA that improves lung function, patient-reported outcomes such as dyspnoea and quality of life, and reduces exacerbations in patients with COPD [3–9]. Olodaterol is a novel LABA that provides 24-h bronchodilation and symptomatic benefits in patients with COPD [10–13]. The combination of tiotropium + olodaterol has been extensively studied in a large Phase III clinical trial programme that demonstrated improvements in lung function and patient-reported outcomes compared to tiotropium monotherapy, with tolerability similar to tiotropium [14–18]. A recent *post hoc* analysis of the TONADO® studies showed that tiotropium + olodaterol significantly improved lung function in Global initiative for chronic Obstructive Lung Disease (GOLD) severity groups 2, 3 and 4, compared to monotherapy, irrespective of whether patients had received prior LAMA or LABA maintenance treatment [19].

The OTEMTO® studies were two replicate, randomised, double-blind, Phase III studies investigating the effects of tiotropium + olodaterol on lung function and quality of life [16]. Unlike the TONADO® trials, OTEMTO® included a placebo arm as well as tiotropium as an active comparator in order to properly understand the effect size of tiotropium + olodaterol on patient-reported outcomes. Overall, tiotropium + olodaterol was superior to tiotropium at improving quality of life as measured by the St George’s Respiratory Questionnaire (SGRQ) and, importantly, the improvement versus placebo was >4 units (the minimum clinically important difference) [16].

The OTEMTO® studies provided the opportunity to study the effectiveness of tiotropium + olodaterol in different COPD subgroups based on lung function (GOLD 2 or 3), GOLD combined assessment (A, B, C or D) and previous treatment focusing on treatment-naive patients (no prior use of LAMAs, LABAs and/or inhaled corticosteroids [ICS]). We, therefore, performed *post hoc* analyses to evaluate the efficacy of tiotropium + olodaterol compared to placebo and tiotropium monotherapy in subgroups of patients defined by GOLD category (GOLD 2–3 and GOLD A–D) and by previous treatment history (treatment naive and baseline ICS use) after 12 weeks of treatment. The aim of this analysis was to understand if the benefits of tiotropium + olodaterol vary according to GOLD categorisation or previous treatment.

## Methods

### Study design

As presented elsewhere [16], OTEMTO® 1 (1237.25; NCT01964352) and 2 (1237.26; NCT02006732) were two replicate, double-blind, placebo-controlled studies. Patients were randomised to one of four treatment arms to receive once-daily tiotropium + olodaterol 2.5/5 μg, tiotropium + olodaterol 5/5 μg, tiotropium 5 μg or placebo, all delivered via the Respimat® inhaler.

### Patients

Patients were included if they were aged ≥40 years with moderate or severe COPD (GOLD 2–3; post-bronchodilator forced expiratory volume in 1 s [FEV_1_] <80 % and ≥30 % of predicted normal), FEV_1_/forced vital capacity <70 % predicted and a smoking history of >10 pack-years. Exclusion criteria included significant disease other than COPD, a history of asthma, COPD exacerbation or symptoms of lower respiratory tract infection within the previous 3 months.

Patients continued their ICS therapy if they were on a stable dose for 6 weeks prior to screening but were not permitted to take LAMAs or LABAs other than study medication. Short-acting muscarinic antagonists were permitted only during the screening period and open-label salbutamol was provided as rescue medication for use throughout the study.

The studies were conducted in accordance with the Declaration of Helsinki, International Conference on Harmonisation Harmonised Tripartite Guideline for Good Clinical Practice and local regulations. Signed, informed consent was obtained from all patients. The studies were approved by the relevant Institutional Review Board/Independent Ethics Committees and competent authorities; full details are included in Additional file 1.

### Outcomes

There were three primary end points in the OTEMTO® studies, all at 12 weeks: SGRQ total score, change from baseline in trough FEV_1_ and change from baseline in FEV_1_ area under the curve from 0 to 3 h (AUC_0–3_). Mahler Transition Dyspnoea Index (TDI) focal score was a secondary end point; responder analyses for SGRQ and TDI focal scores were further end points.

### Assessments

As described in the primary manuscript [16], pulmonary function tests were performed at 1 h pre-dose, 10 min pre-dose, 5, 15 and 30 min post-dose and 1, 2 and 3 h post-dose at baseline and week 12, and at 10 min pre-dose only after 2 and 6 weeks of treatment. The final trough FEV_1_ measurement was taken the day after the week 12 visit (at 23 h and 23 h 50 min post-dose). SGRQ was completed in the clinic at baseline and weeks 6 and 12. At weeks 6 and 12, trained clinic staff conducted the TDI interview, which asks patients about breathlessness compared to baseline.

### Subgroup analysis

For all of the *post hoc* analyses presented here, data from OTEMTO® 1 and 2 were combined to increase the robustness of the analyses. As tiotropium + olodaterol 5/5 μg is the licensed dose, we are only presenting the results of these subgroup analyses for the tiotropium + olodaterol 5/5 μg, tiotropium 5 μg and placebo groups.

Four subgroup analyses were performed: GOLD 2 or 3, GOLD A–D (based on the modified Medical Research Council dyspnoea scale), maintenance treatment naive (no prior use of LAMAs, LABAs and/or ICS) and baseline use of ICS. Results are presented for the three primary end points (FEV_1_ AUC_0–3_ response, trough FEV_1_ response and SGRQ total score), the SGRQ responder analysis, TDI focal score and TDI focal score responder analysis.

For the SGRQ responder analysis, patients were classed as responders if their SGRQ total score improved from baseline to week 12 by ≥4 units. Odds ratios were calculated between groups using a logistical regression including the fixed categorical effect of treatment. A restricted maximum likelihood-based mixed effects model repeated measures approach was used for the analysis of continuous end points, including the fixed categorical effects of treatment, test day and treatment-by-test-day interaction, as well as the continuous fixed covariates of baseline and baseline-by-test-day interaction.

The TDI responder analysis was based on the TDI focal score; patients were classed as responders if they had a value that was ≥1.0 unit.

As these analyses are *post hoc* and performed within subgroups with potentially small sample sizes, they are not powered for statistical comparisons within subgroups; therefore, results are presented as forest plots of treatment differences with corresponding 95 % confidence intervals (CIs). Differences in treatment effects between subgroups are discussed and emphasis is not put on statistical significance within subgroups alone (assessed based on the 95 % CI). No adjustment for multiple comparisons has been performed.

## Results

### Patient disposition and baseline characteristics

**Fig. 1:**
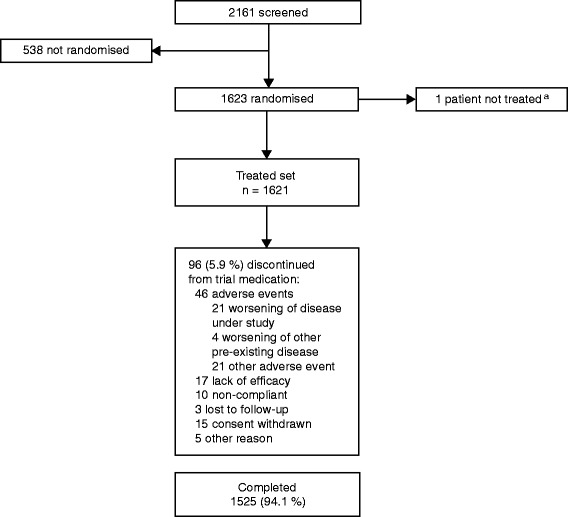
Patient disposition in OTEMTO® 1 and 2 (combined data). ^a^1623 patients were randomised; 1 patient was entered twice but counted only once in the treated set

In OTEMTO® 1 and 2, a total of 1623 patients were randomised, with 1621 receiving treatment and 1525 completing the study (Fig. 1). Patient demographics and baseline characteristics by subgroup and overall population are shown in Table 1. Overall baseline characteristics such as age and sex were similar across subgroups. As expected, mean FEV_1_ values and baseline pulmonary medications also differed between subgroups. Patients who were already receiving maintenance therapy tended to be ex-smokers and to have worse lung function compared to those who were treatment naive.

**Table 1:** Patient demographics and baseline characteristics by subgroups and overall (combined OTEMTO® 1 and 2; treated set)

	GOLD 2	GOLD 3	GOLD A	GOLD B	GOLD C	GOLD D	Naive: no	Naive: yes	ICS: no	ICS: yes	Overall
Patients, n	1042	570	486	483	250	400	943	678	1013	608	1621
Male, n (%)	609 (58.4)	374 (65.6)	298 (61.3)	271 (56.1)	167 (66.8)	250 (62.5)	574 (60.9)	413 (60.9)	632 (62.4)	355 (58.4)	987 (60.9)
Mean (SD) age, years	64.3 (8.7)	65.5 (7.9)	64.3 (8.1)	64.4 (9.0)	65.2 (8.6)	65.4 (7.9)	65.6 (8.2)	63.5 (8.6)	63.9 (8.4)	66.1 (8.3)	64.7 (8.4)
Smoking status, n (%)											
Ex-smoker	554 (53.2)	300 (52.6)	260 (53.5)	255 (52.8)	135 (54.0)	207 (51.8)	560 (59.4)	298 (44.0)	473 (46.7)	385 (63.3)	858 (52.9)
Current smoker	488 (46.8)	270 (47.4)	226 (46.5)	228 (47.2)	115 (46.0)	193 (48.3)	383 (40.6)	380 (56.0)	540 (53.3)	223 (36.7)	763 (47.1)
Mean (SD) pre-bronchodilator											
FEV_1_, L	1.557 (0.460)	0.969 (0.262)	1.617 (0.469)	1.499 (0.445)	1.094 (0.353)	0.988 (0.327)	1.283 (0.459)	1.436 (0.521)	1.422 (0.512)	1.221 (0.428)	1.347 (0.492)
Mean (SD) post-bronchodilator											
FEV_1_, L	1.754 (0.468)	1.146 (0.274)	1.820 (0.472)	1.690 (0.455)	1.269 (0.370)	1.167 (0.336)	1.476 (0.475)	1.619 (0.532)	1.614 (0.522)	1.406 (0.444)	1.536 (0.504)
FEV_1_ % predicted	62.94 (7.98)	41.09 (5.54)	63.66 (8.08)	62.09 (7.72)	44.86 (9.80)	42.58 (9.06)	53.91 (12.86)	56.75 (12.57)	56.65 (12.55)	52.52 (12.83)	55.10 (12.81)
FEV_1_/FVC ratio, % (SD)	54.54 (8.21)	42.82 (9.62)	54.66 (8.33)	54.54 (8.14)	44.13 (9.83)	43.79 (10.24)	48.49 (10.42)	52.87 (9.89)	51.87 (9.96)	47.74 (10.67)	50.32 (10.42)
Baseline pulmonary medication, n (%)											
Any	761 (73.0)	473 (83.0)	349 (71.8)	356 (73.7)	213 (85.2)	321 (80.3)	943 (100)	297 (43.8)	632 (62.4)	608 (100)	1240 (76.5)
ICS	337 (32.3)	269 (47.2)	131 (27.0)	173 (35.8)	125 (50.0)	179 (44.8)	608 (64.5)	0 (0.0)	0 (0.0)	608 (100)	608 (37.5)
LAMA	334 (32.1)	221 (38.8)	157 (32.3)	150 (31.1)	105 (42.0)	148 (37.0)	560 (59.4)	0 (0.0)	277 (27.3)	283 (46.5)	560 (34.5)
SAMA	68 (6.5)	56 (9.8)	24 (4.9)	38 (7.9)	21 (8.4)	41 (10.3)	88 (9.3)	36 (5.3)	55 (5.4)	69 (11.3)	124 (7.6)
LABA	365 (35.0)	261 (45.8)	149 (30.7)	184 (38.1)	117 (46.8)	179 (44.8)	629 (66.7)	0 (0.0)	144 (14.2)	485 (79.8)	629 (38.8)
SABA	470 (45.1)	343 (60.2)	211 (43.4)	223 (46.2)	159 (63.6)	224 (56.0)	542 (57.5)	276 (40.7)	447 (44.1)	371 (61.0)	818 (50.5)

GOLD A–D based on modified Medical Research Council dyspnoea scale. The inclusion criteria for this study only included patients with GOLD 2 or 3 disease; however, 8 patients were classed as GOLD 4 and 1 patient as GOLD 1 based on entrance spirometry results. As this is a small number, these patients were not included in the GOLD 2/3 subgroup analysis and are not included in the table

*GOLD* Global initiative for chronic Obstructive Lung Disease, *ICS* inhaled corticosteroids, *SD* standard deviation, *FEV*_*1*_ forced expiratory volume in 1 s, *FVC* forced vital capacity, *LAMA* long-acting muscarinic antagonist, *SAMA* short-acting muscarinic antagonist, *LABA* long-acting β_2_-agonist, *SABA* short-acting β-agonist

### Efficacy

#### GOLD 2 and 3 subgroups

**Fig. 2:**
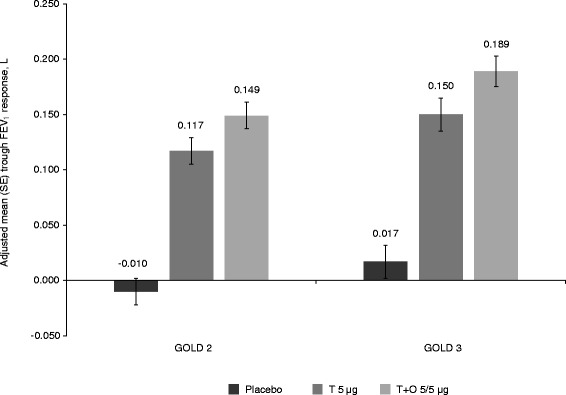
Adjusted mean trough FEV_1_ responses at 12 weeks in patients with GOLD 2 and 3 disease. FEV_1_: forced expiratory volume in 1 s; GOLD: Global initiative for chronic Obstructive Lung Disease; SE: standard error; T: tiotropium; O: olodaterol

**Fig. 3:**
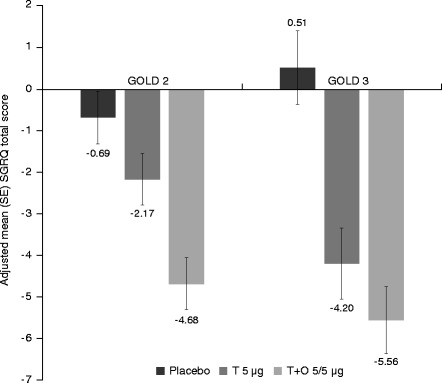
Adjusted mean SGRQ total score at 12 weeks in patients with GOLD 2 and 3 disease. SGRQ: St George’s Respiratory Questionnaire; GOLD: Global initiative for chronic Obstructive Lung Disease; SE: standard error; T: tiotropium; O: olodaterol

Trough FEV_1_ responses and SGRQ total scores improved with tiotropium + olodaterol in both GOLD 2 and 3 subgroups after 12 weeks of treatment compared to baseline (Figs. 2 and 3). In the GOLD 2 subgroup, the adjusted mean (standard error) SGRQ total score change from baseline at week 12 was −4.7 (0.6) in the tiotropium + olodaterol 5/5 μg arm, −2.2 (0.6) in the tiotropium 5 μg arm and −0.7 (0.6) in the placebo arm. In the GOLD 3 subgroup, the adjusted mean (standard error) SGRQ total score change from baseline at week 12 was −5.6 (0.8) in the tiotropium + olodaterol 5/5 μg arm, −4.2 (0.9) in the tiotropium 5 μg arm and 0.5 (0.9) in the placebo arm. Similar improvements were also seen in the absolute SGRQ score (Additional file 1: Table S1). Improvements after 12 weeks compared to baseline were also observed for tiotropium + olodaterol 5/5 μg in the GOLD 2 and 3 subgroups for FEV_1_ AUC_0–3_ response (292–326 mL) and TDI (1.67–1.85) (Additional file 1: Table S1).

**Fig. 4:**
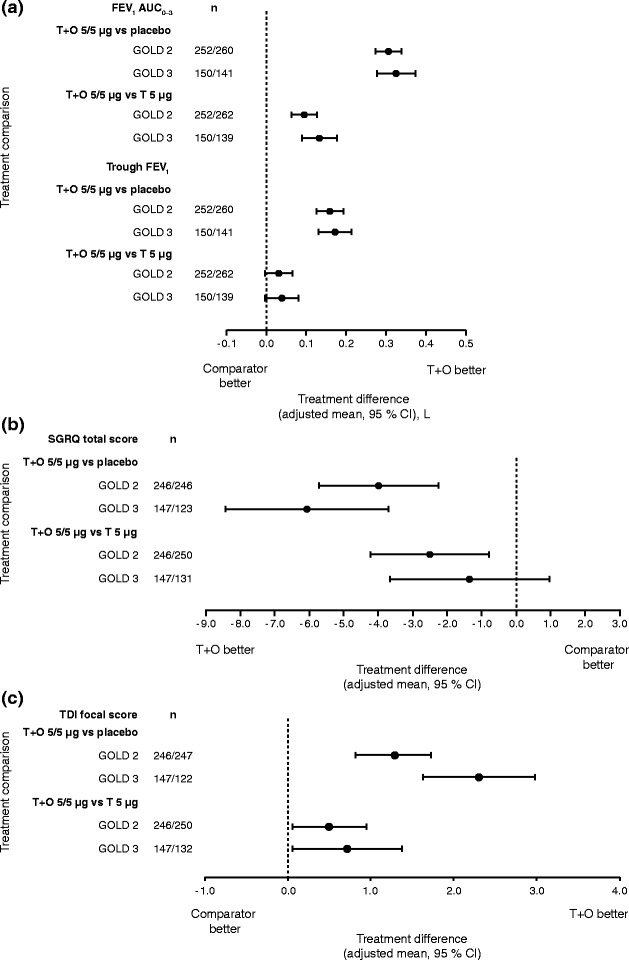
(**a**) FEV_1_ AUC_0–3_ and trough FEV_1_ responses, (**b**) SGRQ total score and (**c**) TDI focal score, all at 12 weeks: treatment comparisons for T + O 5/5 μg versus T 5 μg and versus placebo in patients with GOLD 2 and 3 disease. FEV_1_: forced expiratory volume in 1 s; AUC_0–3_: area under the curve from 0–3 h; SGRQ: St George’s Respiratory Questionnaire; TDI: Transition Dyspnoea Index; T: tiotropium; O: olodaterol; GOLD: Global initiative for chronic Obstructive Lung Disease; CI: confidence interval

Treatment differences are shown in Fig. 4 for FEV_1_ AUC_0–3_, trough FEV_1_ responses, SGRQ total score and TDI focal score. In general, results in GOLD 2 patients were similar to GOLD 3, with larger CIs for GOLD 3 due to the smaller number of patients in this group. The comparison of tiotropium + olodaterol versus placebo showed significant treatment effects for all end points in GOLD 2 and 3 patients (95 % CI did not cross zero). The comparisons of tiotropium + olodaterol versus tiotropium showed significant differences between treatment for most end points, except for trough FEV_1_, in both GOLD 2 and 3, and, as discussed, SGRQ in GOLD 3 patients, which is most likely due to the reduced sample size as treatment differences are similar between the groups.

#### GOLD A–D subgroups

**Fig. 5:**
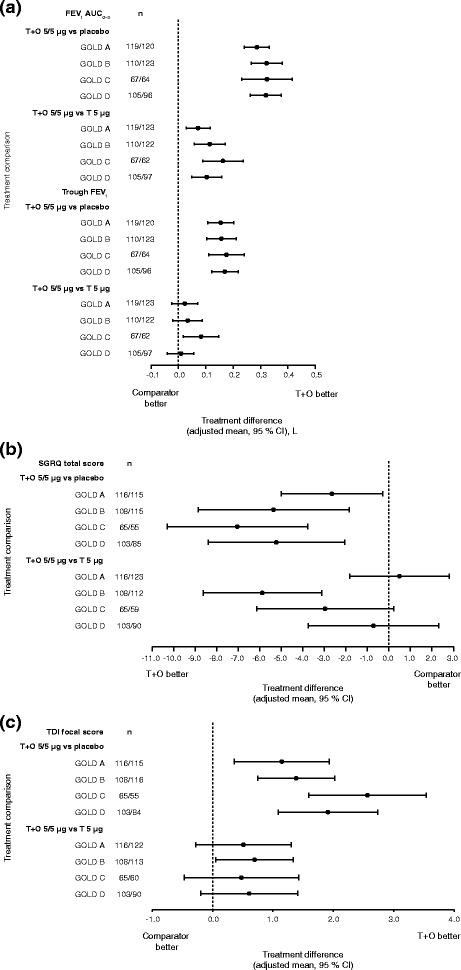
(**a**) FEV_1_ AUC_0–3_ and trough FEV_1_ responses, (**b**) SGRQ total score and (**c**) TDI focal score, all at 12 weeks: treatment comparisons for T + O 5/5 μg versus T 5 μg and versus placebo in patients with GOLD A–D disease. FEV_1_: forced expiratory volume in 1 s; AUC_0–3_: area under the curve from 0–3 h; SGRQ: St George’s Respiratory Questionnaire; TDI: Transition Dyspnoea Index; T: tiotropium; O: olodaterol; GOLD: Global initiative for chronic Obstructive Lung Disease; CI: confidence interval

Tiotropium + olodaterol was more effective at improving FEV_1_ AUC_0–3_ than tiotropium monotherapy and placebo in GOLD A–D subgroups (Fig. 5). Trough FEV_1_ responses improved significantly with tiotropium + olodaterol compared to placebo in GOLD A–D subgroups. Improvements with tiotropium + olodaterol were numerically better compared to tiotropium monotherapy in GOLD A, B and D groups, although these improvements were not significant, as the 95 % CI crossed zero. In general, lung-function treatment comparisons were similar across GOLD A–D subgroups.

SGRQ total score and TDI focal score improved significantly, to a greater extent with tiotropium + olodaterol compared to placebo in GOLD A–D groups (Fig. 5b and c). Treatment effects were similar between subgroups with overlapping 95 % CIs. When comparing tiotropium + olodaterol to tiotropium alone, treatment effects differed between subgroups and improvements in SGRQ total score were most evident in the GOLD B subgroup.

#### Treatment-naive patients

**Fig. 6:**
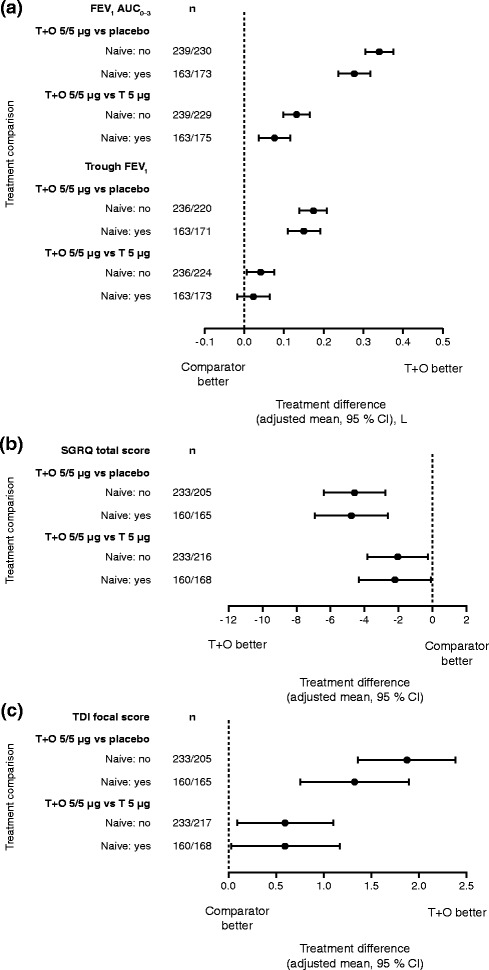
(**a**) FEV_1_ AUC_0–3_ and trough FEV_1_ responses, (**b**) SGRQ total score and (**c**) TDI focal score: treatment comparisons at 12 weeks for T + O 5/5 μg versus T 5 μg and versus placebo in patients who were treatment naive/not treatment naive at baseline. FEV_1_: forced expiratory volume in 1 s; AUC_0–3_: area under the curve from 0–3 h; SGRQ: St George’s Respiratory Questionnaire; TDI: Transition Dyspnoea Index; T: tiotropium; O: olodaterol; CI: confidence interval

Tiotropium + olodaterol was more effective than tiotropium monotherapy and placebo for FEV_1_ AUC_0–3_ and trough FEV_1_, both in patients with and without previous maintenance therapy, although for trough FEV_1_ the 95 % CI crossed zero for the comparison with tiotropium in treatment-naive individuals. The effect sizes were generally greater in those patients who had been receiving previous maintenance therapy (Fig. 6a). The mean improvements in SGRQ total score and TDI focal score were similar for patients with and without previous maintenance therapy and significant for both treatment comparisons (Fig. 6b and c).

#### ICS use at baseline

**Fig. 7:**
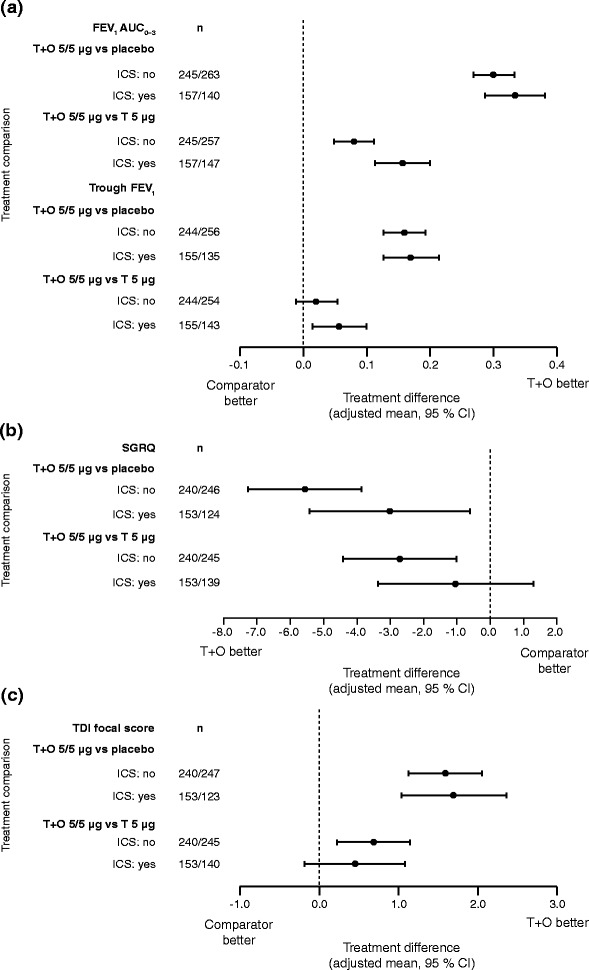
(**a**) FEV_1_ AUC_0–3_ and trough FEV_1_ responses, (**b**) SGRQ total score and (**c**) TDI focal score: treatment comparisons at 12 weeks for T + O 5/5 μg versus T 5 μg and versus placebo in patients who were receiving/ not receiving ICS treatment at baseline. FEV_1_: forced expiratory volume in 1 s; AUC_0–3_: area under the curve from 0–3 h; SGRQ: St George’s Respiratory Questionnaire; TDI: Transition Dyspnoea Index; T: tiotropium; O: olodaterol; ICS: inhaled corticosteroids; CI: confidence interval

Tiotropium + olodaterol was more effective at improving lung-function outcomes than tiotropium monotherapy and placebo, both in patients who were receiving ICS treatment at baseline and those who were not. FEV_1_ AUC_0–3_ and trough FEV_1_ responses improved in both groups (Fig. 7a), with some evidence for a superior response for patients receiving ICS at baseline. Baseline SGRQ scores were higher for patients receiving ICS treatment at baseline, compared to those not receiving baseline ICS (44.4 and 41.5, respectively). In contrast to lung-function outcomes, the improvement in SGRQ total scores with tiotropium + olodaterol compared to tiotropium or placebo was greater in patients who were not receiving ICS at baseline (Fig. 7b and c).

#### Responder analyses across subgroups

Responder analyses for SGRQ total score demonstrated that a greater proportion of patients responded with tiotropium + olodaterol compared to tiotropium monotherapy or placebo for all subgroups (Additional file 1: Figure S1a) with significant improvements compared to placebo in all subgroups except GOLD A. Overall, treatment differences for tiotropium + olodaterol compared to tiotropium monotherapy or placebo were similar between subgroups, with widely overlapping 95 % CIs. In the GOLD 2 subgroup, 52.8 % of patients in the tiotropium + olodaterol 5/5 μg arm and 39.2 % in the tiotropium 5 μg arm were SGRQ responders, compared to 33.7 % in the placebo arm. In the GOLD 3 subgroup, 51.7 % of patients in the tiotropium + olodaterol 5/5 μg arm and 45.0 % in the tiotropium 5 μg arm were SGRQ responders, compared to 28.5 % in the placebo arm. There was also an increased proportion of TDI responders with tiotropium + olodaterol compared to tiotropium monotherapy and placebo in all subgroups, with similar treatment effect overall (Additional file 1: Figure S1b). Improvements were significant compared to placebo in all subgroups and compared to tiotropium for most of the subgroups.

## Discussion

The OTEMTO® studies demonstrated that tiotropium + olodaterol improved lung function and quality of life in patients with moderate to severe COPD. Subgroup analyses confirm that tiotropium + olodaterol is equally effective in patients with moderate COPD (GOLD 2), as for those with severe disease (GOLD 3). Using the GOLD A–D categorisation, tiotropium + olodaterol showed a similar effect on lung function in all the subgroups but the benefit of this dual bronchodilator combination versus tiotropium monotherapy on patient-reported outcomes was most apparent in GOLD B patients. Tiotropium + olodaterol was also superior to tiotropium monotherapy irrespective of previous treatment history, even in those patients naive to all maintenance therapy.

The responder analyses are an alternative way of understanding the treatment effects on patient-reported outcomes. Importantly, there was a greater proportion of SGRQ and TDI responders with tiotropium + olodaterol compared to tiotropium in GOLD 2 patients, demonstrating the potential benefits of tiotropium + olodaterol in terms of quality of life for patients with moderate disease.

*Post hoc* analyses of the TONADO® studies showed that tiotropium + olodaterol was superior to tiotropium and olodaterol monotherapies for lung-function changes in patient subgroups classified by lung-function impairment severity, age and previous treatment history [19]. Our analyses generally agree with these previous findings on lung function and further extend these observations by investigating responses in patients with GOLD A–D disease, as well as investigating patient-reported outcomes. The analysis of patients with GOLD A–D disease showed that the effects of tiotropium + olodaterol on lung function versus tiotropium monotherapy or placebo were similar in all subgroups. However, for SGRQ, the benefit of tiotropium + olodaterol was most apparent in GOLD B patients. There has been some debate about which patients with COPD benefit most from dual bronchodilator treatment compared to long-acting bronchodilator monotherapy. The current findings in GOLD B patients suggest that the symptomatic benefit of tiotropium + olodaterol is greater in patients with higher baseline symptoms and FEV_1_ >50 % predicted and this may indicate that early combined treatment would be beneficial for these patients. The treatment difference was less pronounced in GOLD D patients compared to GOLD B patients, suggesting that in highly symptomatic patients with COPD (*i.e.* GOLD B and D) the greatest benefit of LAMA + LABA compared to long-acting bronchodilator monotherapy on patient-reported outcomes is in patients with less severe airflow obstruction, although all groups showed benefit from LAMA + LABA therapy. A note of caution for this interpretation of the GOLD A–D analysis is that the patients were split into four subgroups, thereby producing the smallest sample size of all the analyses presented here, and this reduced sample size may influence these results.

There was a trend towards greater lung-function improvements with tiotropium + olodaterol in patients who had received previous maintenance therapy. However, this pattern did not translate to a greater benefit on patient-reported outcomes. The mean treatment effects were similar in both groups of patients and the wide 95 % CI for the treatment effect in patients receiving previous maintenance treatment suggests a large variation between individuals in this group, and results may have been influenced by selection bias. Overall, we conclude that treatment with tiotropium + olodaterol showed evidence of similar efficacy, irrespective of previous maintenance treatment.

In this study, there was a large proportion of patients receiving ICS treatment at baseline, the majority of whom were classed as having severe or very severe (GOLD 3 or GOLD C/D) disease. This is in accordance with current GOLD guidelines, which recommend ICS treatment for patients with severe/very severe COPD and frequent exacerbations who are not adequately controlled with long-acting bronchodilators [1]. Clinical trials of LAMA + LABA combinations usually allow patients receiving ICS treatment to continue during the study. This is mostly for ethical reasons, as stepping patients down from both long-acting bronchodilator treatment and ICS in the placebo arm is potentially unsafe. However, the use of LAMA + LABA combination inhalers in real life is likely to be mostly without concomitant ICS treatment. Although this sub-analysis of OTEMTO® cannot exclude an effect of ICS treatment on the clinical response to tiotropium + olodaterol, it would appear that any effect, if present, is small. Further studies are required to better assess whether patients with GOLD D disease should be treated with LAMA + LABA or LAMA + LABA + ICS.

The main limitations of these analyses are that they are *post hoc* and not powered for statistical comparisons between subgroups. However, as the analyses were performed to confirm that the positive results of the OTEMTO® study hold true for patients of all disease severities included, and for patients irrespective of prior medication use, this is not a major concern. A pattern of results for the lung-function changes noted in this subgroup analysis is that often the 95 % CI for tiotropium + olodaterol versus tiotropium crossed zero for trough FEV_1_ but not for FEV_1_ AUC_0–3_. This, again, is likely to be related to sample size for these subgroups and the greater effect size for AUC_0–3_.

One reason why it is of interest to examine the benefits of tiotropium + olodaterol in patients by disease severity, and in patients who are treatment naive, is that the fastest decline in lung function in COPD is seen in the initial stages of the disease [20]. Thus, selecting the most appropriate treatment for initial therapy may be important. Even in mild to moderate COPD, patients start to limit exercise and activities, which are further limited as lung function declines, leading to a sedentary lifestyle [21]. It has been suggested that intervening early in COPD may benefit patients, as they are able to maintain levels of activity and health [20], and treatment may improve health-related quality of life and potentially slow disease progression [22]. Furthermore, a subgroup analysis of the UPLIFT® tiotropium study showed that in patients with GOLD 2 (moderate) COPD, early intervention with tiotropium appeared to slow the course of FEV_1_ decline [23]. Improvements in lung function are associated with better quality of life and reduced symptoms [24]. By targeting earlier disease, it is hoped that treatment may maintain the patient’s functionality and postpone disease progression for longer.

## Conclusions

Overall, the added benefits of tiotropium + olodaterol compared to tiotropium monotherapy in patients with moderate COPD and treatment-naive patients suggest that tiotropium + olodaterol should be considered as an option for patients at the point where there is a need to initiate maintenance therapy, as well as in patients with more severe disease.

## Abbreviations

AUC_0–3_, area under the curve from 0 to 3 h; CI, confidence interval; COPD, chronic obstructive pulmonary disease; FEV_1_, forced expiratory volume in 1 s; GOLD, Global initiative for chronic Obstructive Lung Disease; ICS, inhaled corticosteroids; LABA, long-acting β_2_-agonist; LAMA, long-acting muscarinic antagonist; SGRQ, St George’s Respiratory Questionnaire; TDI, Transition Dyspnoea Index.

## Additional file

Additional file 1: Methodology; **Table S1.** Trough FEV_1_ and FEV_1_ AUC_0–3_ responses, TDI focal score and SGRQ total score at 12 weeks in GOLD 2 and GOLD 3 subgroups (full analysis set, combined studies); **Figure S1.** (a) Responder analysis of SGRQ total score and (b) TDI focal score, all at 12 weeks: treatment comparisons for T + O 5/5 μg versus T 5 μg and versus placebo in patients with GOLD 2 and 3 disease, GOLD A–D disease, patients who were treatment naive/not treatment naive at baseline and for patients who were receiving/not receiving ICS treatment at baseline. (DOCX 333 kb)
