# Supplementary material for: Effects of tiotropium + olodaterol versus tiotropium or placebo by COPD disease severity and previous treatment history in the OTEMTO® studies
Source: Respir Res. 2016 Jun 18;17:73. doi: 10.1186/s12931-016-0387-7 (PMC4912717; doi:10.1186/s12931-016-0387-7)

**Methodology**

The protocols for OTEMTO^®^ 1 and 2 were approved by the individual Institutional Review Boards (IRBs) for each trial centre. The relevant IRBs and centre numbers for each country are listed below.

**OTEMTO^®^ 1**

**Belgium**

- UZ Brussel Commissie voor Medische Ethiek Laarbeeklaan 101, 1090 Brussel (32001).

**Canada**

- IRB Services, 372 Holland Trail, Suite 300 Aurora, Ontario L4G 0A5 (11501, 11502, 11505, 11506, 11507, 11510).
- Health Research Ethics Board of Alberta, Clinical Trials Committee 1500, 10104 – 103 Avenue NW, Edmonton, Alberta, T5J 4A7 (11503, 11504, 11508).

**Czech Republic**

- CEC: Etiska Komise pro Multicentricka Klinicka Hodnoceni, Fakultni Nemocnice v Motole, V Uvalu 84, 15006, Praha 5 (42001, 42002, 42003, 42004, 42005).

**Denmark**

- De Videnskabsetiske, Komitéer for Region Syddanmark Sundhedssekretariatet Damhaven 12, 7100 Vejle (45001, 45002, 45003, 45004).

**Finland**

- Varsinais-Suomen Sairaanhoitopiirin Kuntayhtymän eettinen Toimikunta Kiinamyllynkatu 4-8, 20521 Turku (35801, 35802, 35803).

**Germany**

- Ethikkommission der Ärztekammer Schleswig-Holstein Bismarckallee 8-12 23795 Bad Segeberg (49501, 49502, 49503, 49504, 49505, 49506, 49507, 49508, 49509, 49510, 49511, 49512, 49513, 49514, 49515, 49516).

**Great Britain**

- NRES Committee North West – Greater Manchester Central, 3rd Floor, Barlow House, 4 Minshull Street, Manchester, M1 3DZ (44001, 44002, 44003, 44004, 44005).

**Spain**

- Comité Ético de Investigación Clínica del Hospital Universitario Puerta de Hierro Majadahonda, C\ Joaquín Rodrigo, 2 28222-Majadahonda, Madrid (34001, 34002, 34003, 34004, 34007).

**South Africa**

- UCT Ethics Faculty of Health Sciences, Room E52-23, Groote Schuur Hospital Old Main Building Observatory, 7925 (27501).
- Stellenbosch Ethics Health Research Ethics Committee, Division of Research and Support, PO Box 19063, Tygerberg 7505, 021 938 9677 (27502).
- Pharma Ethics 123 Amcor Road Lyttelton Manor 0157 (27503, 27504, 27505, 27506).

**USA**

- Chesapeake IRB 6940 Columbia Gateway Drive Suite 110, Columbia, MD 21046-3403 (10501, 10502, 10503, 10504, 10505, 10506, 10507, 10508, 10509, 10510, 10511, 10513, 10514, 10515, 10516, 10517, 10518, 10519, 10520, 10521).

**OTEMTO^®^ 2**

**Australia**

- Sir Charles Gairdner Human Research Ethics Committee (61001).
- Southern Adelaide Clinical Human Research Ethics Committee (61002, 61007).
- Bellberry Limited Human Research Ethics Committee (61003, 61005).
- Sydney Local Health District Human Research Ethics Committee – Concord Repatriation General Hospital (61004).

**Austria**

- CEC Ethikkommission des Landes Oberösterreich Wagner-Jauregg-Weg 15 4020 Linz (43001, 43002, 43003, 43004, 43006).
- LEC 43004, Ethikkommission des Landes Steiermark Abt 8, FAGPSanitätsdirektion Amt der Steiermärkischen Landesregierung Friedrichgasse 9/E/28 8010 Graz (43004).

**Canada**

- IRB Services, 372 Holland Trail, Suite 300 Aurora, Ontario L4G 0A5 (11601, 11602, 11603, 11605, 11606, 11607, 11608, 11609, 11611).
- Health Research Ethics Authority Suite 200, 95 Bonaventure Avenue, St John’s NL A1B 2X5 (11604).

**Germany**

- Ethikkommission der Ärztekammer Schleswig-Holstein Bismarckallee 8-12 23795 Bad Segeberg (49601, 49602, 49603, 49604, 49605, 49606, 49607, 49608, 49609, 49610, 49611, 49612, 49613, 49614, 49615, 49616).

**Greece**

- EED Leoforos Mesogion, 284, P.C: 15562, Cholargos, Athens (30001, 30002, 30003, 30004, 30005).

**New Zealand**

- Northern A Health and Disability Ethics Committee (64001).

**Norway**

- Regional Komité for Medisinsk og Helsefaglig Forskningsetikk (REK sør-øst) (47001, 47002, 47003, 47004).

**Slovakia**

- CEC (LEC 42104 site): Eticka Komisia Presovskeho Samospravneho Kraja Urad Presovskeho Samospravneho Kraja Namestie Mieru 2 080 01 Presov (42101, 42102, 42103, 42104).
- LEC: Eticka Komisia Narodny ustav Tuberkulozy, Plucnych Chorob a Hrudnikovej Chirurgie 059 84 Vysne Hagy (42101).
- LEC: Eticka Komisia, Urad Kosickeho Samospravneho Kraja Namestie Maratonu Mieru 1 042 66 Kosice (42102).
- LEC: Eticka Komisia, Nemocnica s, Poliklinikou sv. Jakuba, Bardejov ul. Sv. Jakuba 21, 085 01 01 Bardejov (42103).

**South Africa**

- Pharma Ethics, 123 Amcor Road, Lyttelton Manor, Pretoria (27601, 27602, 27604, 27605).

**Sweden**

- Regionala, Etikprövningsnämnden, i Lund, Box 133, 221 00 Lund (46001, 46002, 46003, 46004).

**USA**

- Chesapeake IRB 6940 Columbia Gateway Drive Suite 110 Columbia, MD 21046-3403 (1061, 1062, 1063, 1064, 1065, 1066, 1067, 1068, 1069, 10610, 10613, 10614, 10615, 10616, 10617, 10618, 10619, 10620, 10621).
- Mercy St. Vincent, Medical Center Adult IRB 2213 Cherry Street Toledo, OH 43608 (10612).

**Results**

**Table S1** Trough FEV_1_ and FEV_1_ AUC_0–3_ responses, TDI focal score and SGRQ total score at 12 weeks in GOLD 2 and GOLD 3 subgroups (full analysis set, combined studies)

| **Treatment** | **Trough**  **FEV_1_, mL** | **FEV_1_**  **AUC_0–3_, mL** | **TDI** **focal score** | **SGRQ total score** |
| --- | --- | --- | --- | --- |
| GOLD 2 |  |  |  |  |
| Common baseline  Placebo  Tiotropium 5 µg  Tiotropium + olodaterol 5/5 µg | 1516 (15) -10 (12) 117 (12) 149 (12) | 1516 (15) -14 (12) 197 (11) 292 (12) | 6.87 (0.06) 0.39 (0.16) 1.17 (16) 1.67 (0.16) | 39.9 (0.5) 39.2 (0.6) 37.7 (0.6) 35.2 (0.6) |
| GOLD 3 |  |  |  |  |
| Common baseline  Placebo  Tiotropium 5 µg  Tiotropium + olodaterol 5/5 µg | 943 (11) 17 (15)  150 (15) 189 (14) | 943 (11) 1 (19) 193 (17) 326 (15) | 5.937 (0.088) -0.45 (0.26)  1.13 (0.25) 1.85 (0.23) | 47.4 (0.7) 47.9 (0.9) 43.2 (0.9) 41.9 (0.8) |

Data are presented as adjusted mean (standard error).
Number of patients contributing to the analysis – trough FEV_1_ and FEV_1_ AUC_0–3_: GOLD 2 placebo n=260, tiotropium 5 µg n=262, tiotropium + olodaterol n=252; GOLD 3 placebo n=141, tiotropium 5 µg n=139, tiotropium + olodaterol n=150. TDI focal score: GOLD 2 placebo n=247, tiotropium 5 µg n=250, tiotropium + olodaterol n=246; GOLD 3 placebo n=122, tiotropium 5 µg n=132, tiotropium + olodaterol n=147. SGRQ total score: GOLD 2 placebo n=246, tiotropium 5 µg n=250, tiotropium + olodaterol n=246; GOLD 3 placebo n=123, tiotropium 5 µg n=131, tiotropium + olodaterol n=147.
FEV_1_: forced expiratory volume in 1 s; AUC_0–3_: area under the curve from 0–3 h; TDI: Transition Dyspnoea Index; SGRQ, St George’s Respiratory Questionnaire; GOLD: Global initiative for chronic Obstructive Lung Disease.

**Fig. S1** (a) Responder analysis of SGRQ total score and (b) TDI focal score, all at 12 weeks: treatment comparisons for T+O 5/5 µg versus T 5 µg and versus placebo in patients with GOLD 2 and 3 disease, GOLD A–D disease, patients who were treatment naive/not treatment naive at baseline and for patients who were receiving/ not receiving ICS treatment at baseline
SGRQ: St George’s Respiratory Questionnaire; TDI: Transition Dyspnoea Index; T: tiotropium; O: olodaterol; GOLD: Global initiative for chronic Obstructive Lung Disease; ICS: inhaled corticosteroids; CI: confidence interval


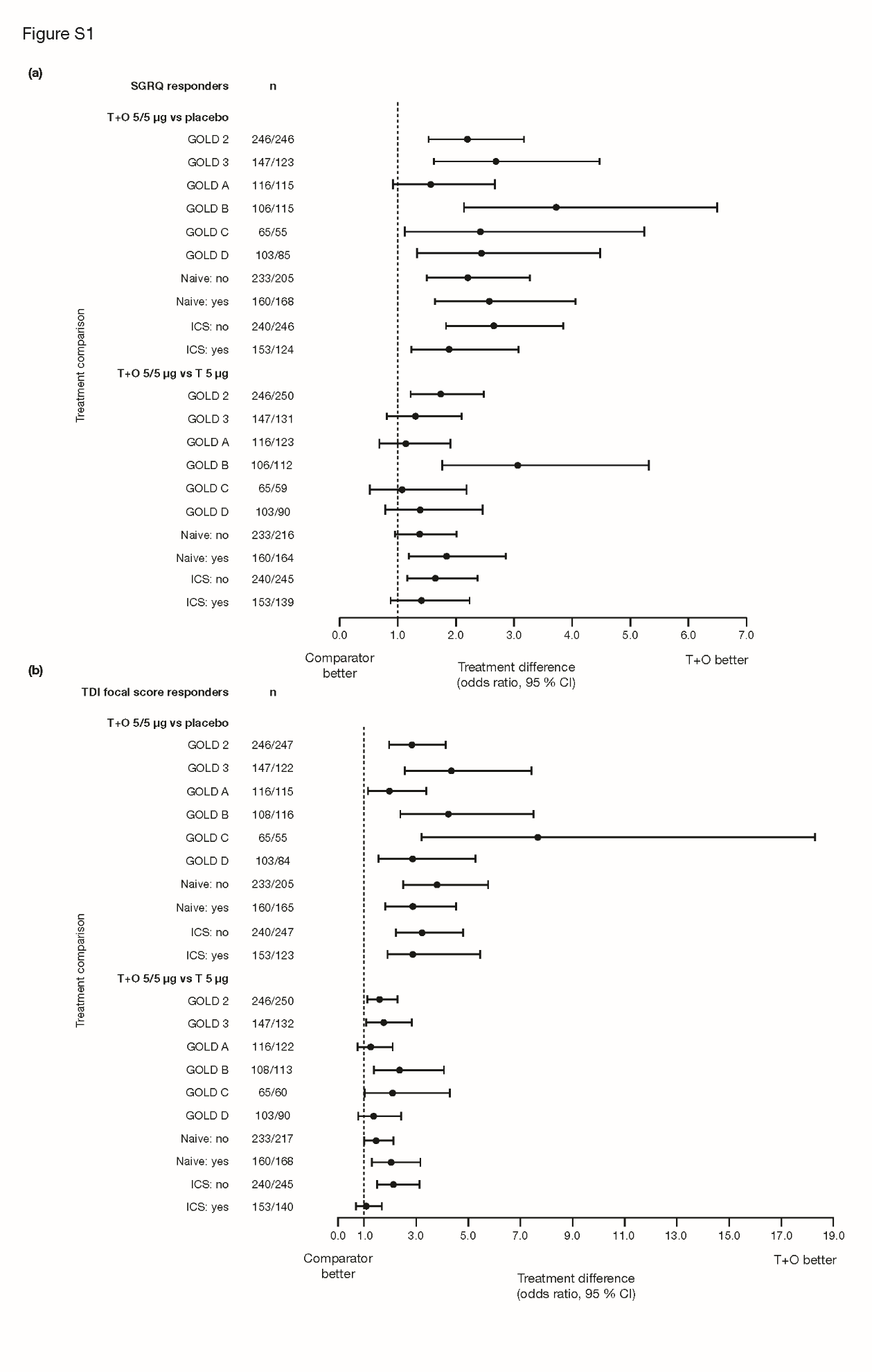

Supplement: Additional file 1: — Methodology; Table S1. Trough FEV1 and FEV1 AUC0–3 responses, TDI focal score and SGRQ total score at 12 weeks in GOLD 2 and GOLD 3 subgroups (full analysis set, combined studies); Figure S1. (a) Responder analysis of SGRQ total score and (b) TDI focal score, all at 12 weeks: treatment comparisons for T + O 5/5 μg versus T 5 μg and versus placebo in patients with GOLD 2 and 3 disease, GOLD A–D disease, patients who were treatment naive/not treatment naive at baseline and for patients who were receiving/not receiving ICS treatment at baseline. (DOCX 333 kb) [file 12931_2016_387_MOESM1_ESM.docx]
